# Vestibular assistance systems: promises and challenges

**DOI:** 10.1007/s00415-015-7922-1

**Published:** 2016-04-15

**Authors:** Jean-Philippe Guyot, A. Perez Fornos, N. Guinand, R. van de Berg, R. Stokroos, H. Kingma

**Affiliations:** Department of Clinical Neurosciences, Service of Oto-Rhino-Laryngology, Head and Neck Surgery, University Hospitals, Rue Gabrielle Perret-Gentil 4, 1211 Geneva 14, Switzerland; Division of Balance Disorders, Department of ENT, Maastricht University Medical Center, Maastricht, The Netherlands

**Keywords:** Vertigo

## Abstract

The handicap resulting from a bilateral vestibular deficit is often underestimated. In most cases the deficit settles gradually. Patients do not understand what is happening to them and have many difficulties to describe their symptoms. They have to consult several doctors with different medical specialties before diagnosis. Once the diagnosis is made there is no biological way to “repair” the deficient vestibular apparatus and vestibular exercises are mildly effective. Attempts have been made to help patients using substitution devices replacing the defective vestibular information by tactile or acoustic cues. Currently, efforts are being made towards the development of a vestibular implant, conceptually similar to the cochlear implant for the rehabilitation of deaf patients. In recent years, several experiments on animal models have demonstrated the feasibility of this project. This paper reports the steps accomplished in human experiments and the main results obtained in our laboratory.

## Introduction

It is estimated that 500,000 patients suffer from a complete bilateral vestibular deficit (BVD) in Europe and the US [1]. This figure is probably underestimated because diagnosis is difficult and sometimes missed. Difficulties start with those faced by patients to describe the symptoms resulting from the functional alteration of a system of which they are not aware, and has multiple roles. In addition to the well known maintenance of posture and vision in dynamic situations, the vestibular system contributes to the regulation of the cardiovascular system [2], bony metabolism [3], sleep [4], respiration [5], spatial orientation, and is linked to the limbic system [6–8]. Specific words exist to describe a hearing impairment, deafness, a visual impairment, blindness, but such words do not exist for vestibular disorders. Therefore, patients can only give a fancy description of their problem, and are often misunderstood by doctors. On the other hand, while doctors may easily understand the handicap associated with blindness or deafness and can easily mimic the situation by closing their eyes or blocking their ears, they cannot inflict to themselves an artificial BVD, even partial.

These difficulties are well illustrated in a survey of 19 patients with BVD. It revealed that the delay between the first symptoms and the diagnosis was greater than 2½ years on average. Furthermore, patients had to consult more than seven doctors with different medical specialties before diagnosis [9]! All patients had undergone an ophtalmological evaluation because they mentioned a blurred vision while walking. They also underwent neurological evaluation because they complained of imbalance. In addition, most of them were referred to a psychiatrist because they were considered to suffer from depression. Some patients underwent also psychological tests since a BVD causes substantial cognitive disorders (i.e., environmental perception, spatial orientation, and self-perception [8, 10]). Finally, some underwent investigations in the search of a metabolic or endocrinologic disorder.

Unfortunately, besides physical therapy which is only mildly effective [11], there are no treatment options available today for patients with BVD. However, since awareness of the significant handicap imposed by this deficit is increasing [12], a number of technical therapeutic alternatives are being explored.

## Substitutive systems

The idea behind sensory substitution devices is to replace the information that should be provided by a defective sensory modality by using sensory information from another functioning sensory modality. In the context of BVD, attempts are made to substitute the missing vestibular information using tactile cues. Patients wear a belt at the waist, fitted with actuators that deliver vibrotactile feedback of increasing intensity on the skin when the subject deviates significantly from the upright position [13, 14]. Other systems attempt to replace missing vestibular information with acoustic cues [15].

## Artificial vestibular apparatus

Another approach is to develop an artificial vestibular apparatus, a “vestibular implant”, which is conceptually similar to the cochlear implant successfully used for the rehabilitation of deaf patients.

In the early 1960s, Cohen and Suzuki showed that it was possible to elicit vestibular responses using electrical stimulation of the vestibular apparatus in monkeys [16]. These early attempts lead to the idea of developing a vestibular implant almost 40 years later [17]. Since this concept emerged, several experiments have confirmed its feasibility in animal models [18–20]. The first human experiments were performed in Geneva, Switzerland, and presented in 2004 at the Barany Society in Paris [21], and published in 2007 [22]. Since then many steps have been accomplished towards the development of an artificial vestibular apparatus for human use [23–26].

The prosthesis is made of external and internal components. The external components include motions sensors fixed on patients’ head, and a processor transforming this information into a pattern of electrical signals. The internal components consist in electrodes implanted in the vicinity of the vestibular nerve branches emerging from the ampulla of the semicircular canals [27, 28] or into the ampulla [29].

## Fundamentals

The experiences to date in animals and humans are limited to the restoration of the function the semicircular canals. No attempt is made yet to restore otolithic function. Of all the vestibular reflexes, the vestibulo-ocular reflex is the easiest to observe and quantify. In consequence, it is the main parameter that has been evaluated in the experiments performed in humans. Measuring the postural control is not part of the evaluation yet but will certainly be necessary in the future.

The vestibular system works in a “push–pull” configuration in which information gathered from both ears is used to encode motion in the three dimensional space. For example, for the horizontal semicircular canal a head rotation in the direction of the canal (i.e., rightwards for the right ear and leftwards for the left ear) will result in an increase of the firing rate in the nerve. Conversely, a horizontal head rotation in a direction opposite to the canal (i.e., leftwards for the right ear and rightwards for the left ear) will result in a decrease of the firing rate. This motion-controlled modulation of the spontaneous firing rate results in a compensatory horizontal eye movement in the direction opposite to the head movement. Therefore, a prerequisite to reestablish bi-directional eye movements with unilateral electrical stimulation (i.e., a unilateral vestibular implant) is to restore an artificial “spontaneous” or “baseline” firing rate. This “baseline” activity can then be increased (up-modulated) for generating eye movements in one direction and decreased (down-modulated) for generating eye movements in the opposite direction, according to the direction and velocity of head movements and thereby to generate vestibular reflexes.

## Results in humans

### Patients and surgery

**Fig. 1:**
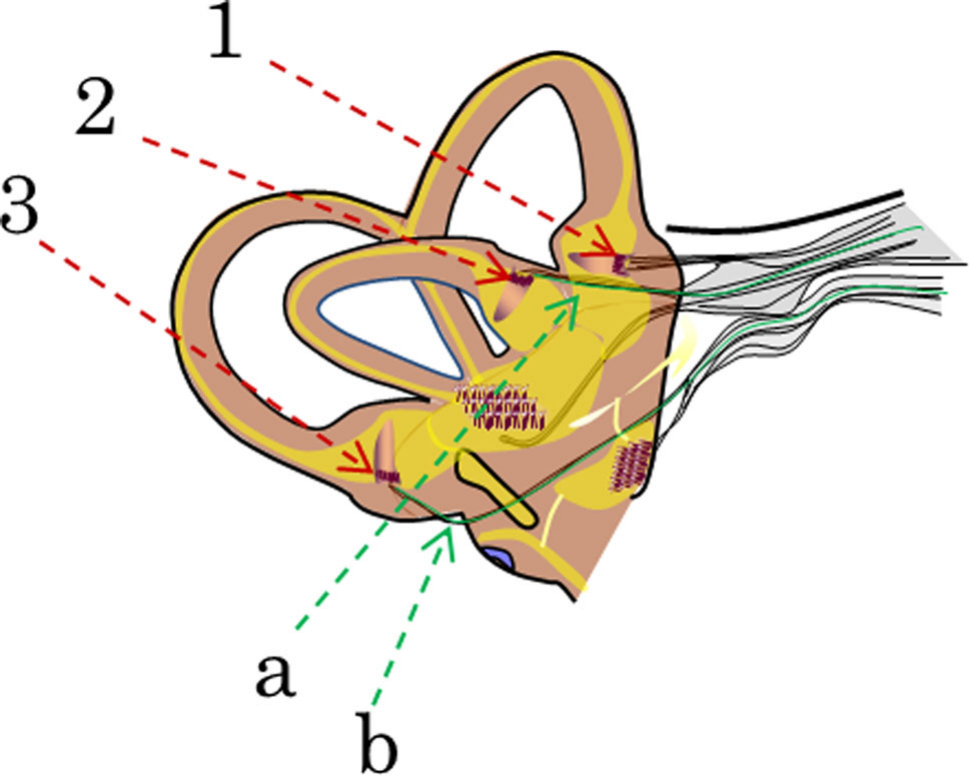
Diagram of the vestibular apparatus. The extralabyrinthic surgical approach consists in placing the electrodes in contact with the branches of the vestibular nerve (in *green*) that emerge from the ampulla of the **a** posterior, and **b** lateral semicircular canals. In the intralabyrinthic approach, the electrodes (in *purple*) are placed in the ampulla of the three semicircular canals

Our research group has developed a prototype vestibular implant based on a commercial cochlear implant in collaboration with Med El© (Innsbruck, Austria). One to three electrodes were taken out of the cochlear array and put in separate electrode leads to be implanted in the vicinity of the posterior, lateral and superior ampullary nerves (see Fig. 1).

Today 12 patients with total BVD have received our prototype vestibular implant. Inclusion criteria were: (1) mean peak slow phase velocity of ≤5°/s in bilateral bithermal caloric irrigations (30° and 44°); (2) pathological Head-Impulse-Test for all six semicircular canals (Synapsis©, Marseille, France; EyeSeeCam VOG©, Munich, Germany; and/or the ICS Otometrics, Denmark); and (3) low (<0.2) or no gain at clinical rotatory chair tests (0.05–0.1 Hz, *ω*_max_ = 60°/s) [see 30]. In addition, since there are non-negligible risks of hearing loss related to the surgical procedure, candidates also had to be severely or profoundly deaf in the implanted ear.

In five patients electrodes were placed close to the vestibular nerve branches emerging from the ampulla of the semicircular canals. In four patients a single electrode was placed close to the posterior ampullary nerve [27], and in one an additional electrode was placed close to the lateral ampullary nerve [28]. This ‘extralabyrinthic’ surgical approach has been developed to limit the risk of hearing loss resulting from the insertion of a foreign body into the inner ear. In addition, it is feasible via the external auditory canal under local anesthesia allowing the observation of nystagmic responses during preoperative electrical stimulation trials to optimize electrode placement. Once the optimal position of the vestibular electrodes is determined, the cochlear array is inserted into the cochlea via a conventional retroauricular approach and a posterior tympanotomy under general anesthesia. Then, the vestibular electrodes are placed at the previously determined sites and secured with artificial cements.

In seven patients an intralabyrinthic surgical approach was performed with one electrode in each of the three ampulla [29]. This is a similar approach to that used in animal experiments, and easier to perform than the extralabyrinthic approach. On the counterpart, it might involve higher risks of inducing hearing loss. Furthermore, since the whole procedure is performed in general anesthesia, only a tonic eye deviation is detectable during the intraoperative electrical stimulation trials.

### Electrical stimulation

During all the experiments presented hereafter, the cochlear electrodes were turned off and stimulation was delivered to each vestibular electrode separately (i.e., one vestibular electrode at a time).

A custom Matlab© program was written to drive the cochlear implant via the Med-El© Research Interface Board II© (RIBII) interface. Stimulation consisted of trains of charge-balanced biphasic pulses (cathodic-first, 400 μs/phase) delivered at 200 pulses per second. To reduce vestibular symptoms related to device activation [24], current amplitude was progressively increased by steps of 10–50 µA. Either a change in nystagmus slow phase velocity (>2°/s) or any “vestibular” sensation reported by the patient was considered as the vestibular threshold. The current level at which any unpleasant phenomenon occurred (i.e., pain or facial nerve stimulation) was chosen as the upper comfortable level. These two limits determined the available dynamic range for stimulation which was established separately for each of the electrodes.

The amplitude level for “baseline” stimulation (see above) was arbitrarily chosen in the middle of the measured dynamic range. This constant-amplitude electrical stimulation was maintained until the disappearance of any vestibular signs or symptoms. The amplitude of the stimulating pulse train could then be modulated to elicit bi-directional eye movements. Eye movements are recorded using a fast monocular 2D video oculography system (EyeSeeCam VOG; Munich, Germany).

### Observations and comments

At first, we confirmed the previous observations in animals that nystagmic responses could be generated by electrical stimulation. We were able to successfully elicit eye movement responses in 24 out of the 27 implanted electrodes in 12 patients. The range of eye velocities elicited via electrical stimulation was variable from patient to patient. However, it is worth noting that the velocity of the elicited eye movements was within the range of compensatory eye movements measured during walking or running [31, 32] and shows similar frequency-dependent behavior to the “natural” vestibule-ocular reflex [26]. These characteristics are, of course, essential for the clinical benefit expected from an artificial vestibular system.

Electrically elicited eye movements were in the plane of the stimulated semicircular canal [22, 23] with minimal deviation from the expected axis for the posterior ampullary nerve (i.e., vertical), and somewhat larger for the lateral semicircular canal [30]. This is likely due to current spread because of the anatomical proximity of the lateral and superior canals. It is expected that the plasticity of the brain will correct this misalignment as already observed during chronic stimulation trials in animal models [33–35]. Additional technical developments, such as improvement of electrodes and surgical techniques or signal processing strategies [36] could also be implemented to minimize response misalignment if necessary.

Our experiments in humans began with an unexpected observation. It is well known that a sudden unilateral loss of the vestibular function causes a severe vertigo with deviation towards the deficient side and a spontaneous nystagmus of opposite direction. It was expected that an abrupt restitution of a baseline electrical activity in the vestibular system would cause the same signs and symptoms, but of opposite direction, as shown in deafferented animal models. In these experiments, the nystagmus towards the side of stimulation lasted several hours [37]. Surprisingly, the time of adaptation of our patients was dramatically shorter, and did not exceed 30 min [24, 38]! In addition, symptoms could be significantly reduced and even eliminated using progressive intensity increases, instead of abrupt stimulation onsets. When the stimulation was abruptly stopped, the time of adaptation was even shorter than for activation, not exceeding 3 min! This observation is of high clinical relevance, since it demonstrates that it will be possible for patients to switch on and off the vestibular prosthesis several times a day (i.e., for a bath, a shower, at night, etc.) without great discomfort. Consequently, there seems to be no need to develop a system providing a continuous electrical power supply, or a waterproof device, making the implementation of the vestibular implant easier than expected.

In experiments made so far, the cochlear implant was shut down during the vestibular stimulation and the vestibular electrodes were stimulated one after the other, and never all at once. By using the electrode implanted in the lateral semicircular canal, we have restored a vestibular-ocular reflex with a gain close to normal by modulating the baseline electrical activity via an inertial sensor [25]. To the best of our knowledge, this was the first demonstration of the feasibility to restore at least partially the vestibular function in the human.

Our preliminary results make us optimistic about the possibility to develop a vestibular implant that is useful for the rehabilitation of patients suffering from BVD. However, several important prerequisites must still be fulfilled. In our experimental patients, the motion sensor is maintained by a simple headband or helmet. We are aware that minimizing the sensory misalignment might reduce the learning time for the brain. Therefore, in the future, it will be necessary to fix the sensor more firmly, for example by a titanium screws implanted in a skull bone. A tight fixation will facilitate adjustment of the processor to obtain accurate alignment with head coordinates. For this purpose, a recently reported calibration method to align gyroscope measurements with the anatomical coordinate system will be used [39].

Many patients with BVD have normal hearing. We must therefore determine the risk of hearing loss associated with the placement of vestibular electrodes. The results of first implantation attempts in humans with Menière’s disease performed at the University of Washington are not very encouraging in this respect [40]. However studies carried out in animal models have demonstrated that it might be possible to develop safer surgical techniques allowing for the preservation of hearing [41]. It is therefore imperative to find strategies to minimize the risk of hearing loss and find ways to restore hearing whenever deafness occurs following implantation. One possibility is to develop a total inner ear prosthesis with both functions, vestibular and cochlear, similar to our prototype. The challenge will then be to stimulate all the electrodes simultaneously, cochlear and vestibular without interaction between the two types of stimulation. The actual clinical benefit experienced by patients by the restitution of the sole semicircular canal function without otolithic function also remains to be determined.

Moreover, it will be necessary to compare the benefit of vestibular implant to that obtained with substitutive systems. Recently, a positive effect of galvanic vestibular stimulation on postural control in patients with a bilateral vestibular loss has been observed [42]. These two approaches could be particularly effective for postural control, and the vestibular implant for the control of the VOR. However, it is hoped that with the refinement of the vestibular implant, it will be possible to mimic the natural system ever more precisely, and thus achieve better results than with any other systems. At a minimum the vestibular implant will expand treatment options, and allow offering the most suitable approach to each patient, tailored to his complaints.

## Conclusion

Results up to date strongly support the feasibility of a vestibular implant to rehabilitate patients with BVD. However, it is still premature to say how long it will take to obtain a vestibular implant for clinical use.
